# Methods and Baseline Characteristics of Two Group-Randomized Trials With Multiracial and Multiethnic Working-class Samples

**Published:** 2005-09-15

**Authors:** Anne M Stoddard, Nancy Krieger, Martha E Fay, Elizabeth M Barbeau, Gary G Bennett, Glorian Sorensen, Karen Emmons

**Affiliations:** New England Research Institutes; Department of Society, Human Development and Health, Harvard School of Public Health, Boston, Mass; Department of Society, Human Development and Health, Harvard School of Public Health, Boston, Mass; Department of Society, Human Development and Health, Harvard School of Public Health, and Center for Community Based Research, Dana-Farber Cancer Institute, Boston, Mass; Department of Society, Human Development and Health, Harvard School of Public Health, and Center for Community Based Research, Dana-Farber Cancer Institute, Boston, Mass; Department of Society, Human Development and Health, Harvard School of Public Health, and Center for Community Based Research, Dana-Farber Cancer Institute, Boston, Mass; Department of Society, Human Development and Health, Harvard School of Public Health, and Center for Community Based Research, Dana-Farber Cancer Institute, Boston, Mass

## Abstract

**Introduction:**

Few papers address the methodological challenges in recruiting participants for studies of cancer prevention interventions designed for multiracial and multiethnic working-class populations. This paper reports the results of the sample selection and survey methods for two group-randomized intervention studies.

**Methods:**

The two group-randomized intervention studies, Healthy Directions–Small Business (HD–SB) and Healthy Directions–Health Centers (HD–HC), included a worksite-based study in 26 small manufacturing businesses and a study in 10 outpatient health centers. We used selection and recruitment methods to obtain a multiracial and multiethnic working-class study sample. In 2000 and 2001, we assessed baseline measures of sociodemographic characteristics and behavioral outcomes by self-report. We then computed intraclass correlation coefficients (ICCs).

**Results:**

Of the 1740 participants in the HD–SB study, 68% were non-Hispanic whites, and 76% had working-class occupations. In the HD–HC study, 59% of 2219 participants were non-Hispanic whites. Among those who worked, 51% had working-class occupations. Large percentages of both samples reported not meeting recommended guidelines for the target behaviors. For example, 86% of members of both samples consumed fewer than the recommended five servings of fruits and vegetables per day. The ICCs for the four target behaviors in HD–SB were between 0.006 and 0.02. In the HD–HC study, the ICCs ranged from 0.0004 to 0.003.

**Conclusion:**

The two studies were successful in recruiting multiracial and multiethnic working-class participants. Researchers will find the estimates of the primary outcomes and their ICCs useful for planning future studies.

## Introduction

Increasingly, there have been calls for reducing health disparities based on socioeconomic position and race and ethnicity (1) and for implementing community interventions that address segments of the population in which risk for chronic disease is concentrated (2,3). Few papers in the literature, however, address the methodological challenges in recruiting participants for studies of such interventions. This paper describes and presents the results of the sample selection and survey methods for two group-randomized trials of cancer prevention interventions designed for multiracial and multiethnic working-class populations.

The Harvard Cancer Prevention Program Project, Healthy Directions, was designed to develop and evaluate cancer prevention interventions for multiracial and multiethnic working-class populations (4). The project comprised two intervention studies, Healthy Directions–Small Business (HD–SB) and Healthy Directions–Health Centers (HD–HC), and a cancer prevention policy model-analysis project. The intervention projects were group-randomized controlled studies that tested the shared primary hypotheses that mean levels of dietary and physical activity outcomes would improve more significantly in the intervention group than in the control group. The interventions developed for the two projects were based on a common conceptual framework (4) drawing on social ecological theory (3,5). Using this framework, the social context in which people live was incorporated into the design and delivery of the interventions. This framework encompasses several factors, including individual factors (e.g., material circumstances), interpersonal factors (e.g., family roles and responsibilities), organizational factors (e.g., access to health care), and community factors (e.g., neighborhood safety). In contrast to interventions designed for a specific racial or ethnic group, we used this framework to design interventions that were suitable for a multiracial and multiethnic population.

HD–SB was a worksite-based intervention study designed to test the effectiveness of an integrated health promotion and occupational health protection intervention in 26 small manufacturing businesses in Massachusetts (6). HD–HC was a health-center–based intervention in 10 community health centers in metropolitan Boston (7). The two intervention studies were aimed at four primary outcomes: increasing fruit and vegetable consumption, decreasing red meat consumption, increasing daily multivitamin use, and increasing physical activity. In both studies, the organization was the unit of randomization and intervention, and the individual worker or health center member was the unit of observation.

Group-randomized trials are those in which groups of individuals are randomized to study conditions, but observations are made on the individuals within the groups (8). An advantage of this design is the ability to enhance the intervention effectiveness through the social interactions among members of the groups randomized. The main disadvantage is the loss of statistical efficiency due to the correlation in behavior among members of the same group (9). This study design has been increasing in popularity over the last 25 years, especially for the evaluation of community-based interventions (10). Planning for such studies requires estimates of the within-group correlation of the proposed outcome measures, yet published estimates for specific behaviors and populations are hard to find because there are few publications that include these values in reports of results.

This report focuses on our success in recruiting multiracial and multiethnic working-class participants. We compare the characteristics of the participants with selected characteristics of the larger population within which they reside, and we provide point estimates of the outcome measures and estimates of the intraclass correlation coefficients (ICCs). The ICC is the fraction of the total variation in a measure that is attributable to the clustering of the behavior by members of the same group in comparison with members of different groups (i.e., the health center or worksite). This information is important for researchers planning group-randomized trials in diverse working-class populations.

## Methods

The methods of both studies were approved by Dana-Farber Cancer Institute's Office for the Protection of Research Subjects and the Harvard School of Public Health's Human Subjects Committee. Additionally, the methods of the small business study were approved by Beth Israel Deaconess Medical Center's Committee on Clinical Investigations, and the methods of the health centers study were approved by Harvard Vanguard Medical Associates–Department of Ambulatory Care and Prevention.

### Study populations

#### Small Business

For HD–SB, we identified 224 worksites through D&B (The D&B Corp, Short Hills, NJ; www.dnb.com) listings of manufacturing businesses with Standard Industrial Classification (SIC) codes 20–39 (U.S. Department of Labor, Occupational Safety & Health Administration, Washington, DC; www.osha.gov/pls/imis/sicsearch.html) located in the metropolitan Boston area and employing between 30 and 150 workers. Businesses with these SIC codes were selected because they are more likely than those in other sectors to use potential carcinogens in work processes and thereby are suitable for cancer prevention interventions that integrate health protection and health promotion.

Further eligibility criteria included: 1) employing a multiracial and multiethnic population, defined as 25% of workers being first- or second-generation immigrants or people of color; 2) having an employee turnover rate of less than 20% in the previous year; and 3) being autonomous in decision-making power to participate in the study if part of a larger parent company. Of the 224 businesses initially identified, 197 (88%) completed the prerecruitment survey assessing these eligibility criteria and, of these, 131 (66%) met the criteria.

Finally, companies had to consent to being randomized to receive the behavioral and occupational health intervention and to provide time at work for employees to complete assessment surveys and to participate in the intervention activities. Of the 131 eligible companies, 26 (20%) consented to participate in the study. Details of the recruitment process and comparison of worksites recruited and not recruited are provided elsewhere (11).

Worksites ranged in size from 32 to 137 workers. All employees who met the following criteria were eligible to receive the interviewer-administered survey: 1) permanent employee, 2) worked 20 hours or more per week, 3) worked onsite, and 4) spoke English, Spanish, Portuguese, or Vietnamese. Interviews were conducted in English, Spanish, Portuguese, and Vietnamese between May and December 2000. Of 2096 eligible employees, 1740 (83%) completed the survey.

#### Health Centers

Harvard Vanguard Medical Associates, a 14-center multispecialty medical group practice serving more than 270,000 patients in the greater Boston area, provided the venues for the HD–HC study. We selected the 10 health centers with the most racial, ethnic, and socioeconomic diversity for this study. A random sample of health center members was selected from each center using a list of eligible patients and a random number generator. Eligibility criteria included: 1) living in an eligible neighborhood (see below); 2) being 18 to 75 years old; 3) having a well-care or follow-up visit scheduled with a participating provider; 4) being able to speak and read either English or Spanish (unlike the worksites, Portugese and Vietnamese were not commonly spoken languages); 5) not having cancer at the time of enrollment; and 6) not being employed by the participating health centers or a worksite participating in the small business study. Eligible neighborhoods were defined as census block groups that were predominantly working class (66% or more of employed persons are in working-class occupational groups comprised predominantly of nonsupervisory employees); or met the federal definition of a "poverty area" (20% or more of the population lives below the poverty line); or had low levels of education (25% or more of the adult population has not completed high school) (12).

All 117 providers (physicians, nurse practitioners, and physician assistants) practicing in the internal medicine departments of those centers were approached for permission to recruit from among their patients. A total of 97 (83%) of the 177 clinicians participated, with no differences in the rates of clinician participation between the intervention and control conditions.  

We identified patients in the eligible age range who were scheduled for appointments with one of the participating providers through the health center's automated central appointment system. To determine whether a potential participant lived in an eligible neighborhood, the residential address was geocoded to the census block group, a subdivision of the census tract and the smallest census geographic area (approximately 1000 people) that provides socioeconomic data. Socioeconomic data from the 1990 Census were used to identify eligible neighborhoods. Geocoding was conducted by a commercial firm with verified high accuracy (96%) (13).

Potential participants received a letter describing the study and providing a number to call if they did not want to participate. Members who did not reply within 2 weeks were then contacted by telephone, and after their eligibility was confirmed, they were invited to participate. If they consented, they completed the oral survey at that time or made an appointment to be interviewed by telephone at another time. Study staff attempted to recruit 8963 potentially eligible candidates during 2000 and 2001. Of these, 2547 were unreachable. Among the 6416 who were reached, 867 (14%) were ineligible; 3330 (52%) refused to participate and 2219 (35%) were enrolled. Assuming that 14% of those unreachable were also ineligible, the response rate is 29% of those assumed eligible.

### Measures

Each survey included a core set of items in addition to items unique to that project which reflected mediating and moderating variables.

#### Sociodemographic Characteristics

We assessed three dimensions of socioeconomic position (education, poverty status, and occupational class) and two dimensions of race and ethnicity (racial or ethnic identification and whether the respondent and his or her parents were born in the United States). Respondents reported their educational level in nine categories, which we subsequently collapsed to four (did not complete high school, high school diploma or equivalent, some post-high-school training, and baccalaureate degree or more). Household income was assessed in $10,000 increments from less than $10,000 per year to $50,000 per year or more. We combined the responses to this item with number of people supported by the income and the ages of household members to categorize respondents according to the federal poverty guidelines for food aid (14). In 2001, the poverty guideline for a single person was $9,214; for a family of two adults and two children it was $17,960. The guideline for eligibility for food stamps and The Special Supplemental Nutrition Program for Women, Infants, and Children (WIC) is no more than 185% of the poverty guideline. Respondents were classified as below the poverty guideline, above the poverty guideline but below 185% of the guideline, or above 185% of the poverty guideline. 

We combined information about the respondent's current or most recent job title into a three-category occupational class variable: working class (clerical, sales, skilled or unskilled labor), professional/managerial (professional, managerial, or technical), or no job title. This latter group included health center participants who were homemakers, disabled, and others who were not in the paid labor force and did not report a recent job title.

Participants were asked whether they were of Hispanic or Latino heritage and whether they belonged to any of the four racial groups. We coded participants who reported being of Hispanic or Latino origin in the Hispanic group regardless of any other responses. For the rest, those who reported only one racial group were categorized in that group (i.e., American Indian or Alaska Native, Asian or Pacific Islander, black or African American, or white). Respondents who selected more than one racial group were classified as multiple heritage and were subsequently classified as those who included white and those who did not.

We combined information about the participants' and their parents' birth places into the following three-category measure of immigration status: participant born outside the United States (defined as outside the 50 states and the District of Columbia), participant born in the United States but one or more parents born outside the United States, and participant and both parents born in the United States. Respondents were also asked their birth date and sex.

#### Health Behaviors

The target levels of the health behaviors, based on well-established recommendations (1,15,16), were: five or more servings of fruit and vegetables per day, three or fewer servings of red meat per week, daily multivitamin use, and at least 2.5 hours of moderate or vigorous physical activity per week. For each of the target behaviors we dichotomized the continuously scaled summary measures at the intervention target level so that we could compute the percentage of participants who met the intervention target.

Servings of fruits and vegetables consumed per day were assessed using a screener (17-19) that asked about usual consumption over the last 4 weeks of seven common foods (orange and grapefruit juice, other fruit juice, green salad, fried potatoes, potatoes other than fried, fruit, and other vegetables). For each food, respondents chose 1 of 10 precoded responses from never to five or more times per day. The responses were recoded to equivalent servings per day and summed to obtain total fruit and vegetable servings per day. We then computed a dichotomous measure of either five or more servings per day or less than five servings per day.

Servings of red meat were assessed using an abbreviated form of the semiquantitative food frequency questionnaire (20). The screener asked about usual consumption over the last 4 weeks of six common foods (processed meat; hamburger; beef, ham, pork, or lamb in a sandwich or mixed dish; 4 to 6 ounces of beef, ham, pork, or lamb as a main dish; 4 to 6 ounces of poultry; and 3 to 5 ounces of fish). The six response categories ranged from never to one or more times per day. The responses were recoded to equivalent servings per week and summed for total servings of red meat per week. The totals were dichotomized to three or fewer servings or more than three servings per week.

We based our physical activity assessment on the questionnaire used in the Nurses' Health Study (21). We asked how often on average in the last four weeks respondents engaged in each of eight moderate or vigorous leisure activities. We adapted the items to include specific activities that might be more common in the study population. Activities included walking for exercise; jogging; running; bicycling; aerobics or aerobic dancing; lifting weights; playing soccer, rugby, basketball, lacrosse, baseball, or football; or other activities that get the respondent out of breath. There were eight response categories ranging from never to more than 6 hours per week. In addition, we asked about usual walking pace. The responses were recoded to equivalent minutes per week and summed for total minutes of physical activity per week. Walking was included if usual pace was reported to be faster than "easy, casual." The sum was collapsed to 150 minutes (2.5 hours) or more per week or more compared with fewer than 150 minutes per week.

Furthermore, we asked respondents on average how many days they take a multivitamin. Respondents were coded as taking a multivitamin daily if they reported taking one 6 or 7 days per week.

### Data analysis

For each study sample, we report the number and percentage of participants according to the measures of sociodemographic characteristics and their levels of health behaviors. For comparison purposes, we also present available 2000 census data for the consolidated metropolitan statistical area (CMSA) covering eastern Massachusetts (22). We present the sex distribution for the population aged 18 years and older, educational attainment for the population aged 25 years and older, occupational class for the employed population aged 16 years and older, and percentage below the poverty line for individuals aged 18 years and older. We report race and ethnicity (Hispanic and non-Hispanic white) and percentage of non-U.S.–born for the population as a whole.

For each health behavior, we computed the adjusted percentage of respondents who practice the behavior, controlling for the clustering of participants in randomization units, health centers, or worksites. We also computed the ICC of each health behavior in each study. The adjusted percentages and ICCs were computed using linear logistic regression analysis with group (health center or worksite) as a random effect (8).

Computations were carried out using the GLIMMIX macro to the SAS statistical software (SAS Institute Inc, Cary, NC) (23,24).

## Results

### Sociodemographic characteristics of the two samples

Table 1 shows the sociodemographic characteristics of the two samples and of the greater Boston area. The population of the eastern Massachusetts CMSA is 81% non-Hispanic white, compared with 68% of the HD–SB sample and 59% of the HD–HC sample. The HD–SB sample included 13% Hispanic or Latino ethnicity and approximately equal percentages of blacks (5%) and Asians (7%). In the HD–HC sample, 26% were black and 8% were Hispanic. About one third (34%) of the HD–SB participants and 22% of the HD–HC participants were born outside the United States. Additionally, 10% of the U.S.-born HD–SB participants and 18% of the U.S.-born HD–HC participants had a parent or parents who were born outside the United States.

In the eastern Massachusetts CMSA, 41% of the adults have a high school education or less, slightly less than those in the HD–SB sample (46%) but more than those in the HD–HC sample (28%). Only 24% of the HD–SB participants were professional, managerial, or technical workers. The remaining 76% were employed in working-class occupations (i.e., clerical, sales, skilled or unskilled labor). Among HD–HC participants, approximately equal percentages were employed in professional, managerial, or technical positions (45%) and in working-class occupations (44%). In the greater Boston area, 57% of employed adults are employed in working-class occupations. Although most of the participants in both studies were at or above 185% of the poverty guideline, 15% of HD–SB participants were below this cut point, even though they were all employed. In the HD–HC sample, 18% of participants were below 185% of poverty.

At baseline, most participants in both studies did not meet the intervention targets for fruit and vegetable consumption and daily multivitamin use (Table 2). In the HB–SB study, most participants did not meet the target for red meat consumption, but among HB–HC participants, almost half met that target. Surprisingly high percentages (73% for HB–SB and 65% for HB–HC) of participants reported at least 2.5 hours of physical activity per week in both studies.

### Estimates of ICCs for the primary outcomes

Table 3 presents the adjusted prevalence of each health behavior controlling for the clustering of respondents in randomization units, along with the ICC. The adjusted prevalences of the target behaviors are very close to the unadjusted prevalences presented in Table 2. The ICCs for the four target behaviors in HD–SB were between 0.006 and 0.02, indicating a small level of concordance among workers in the same worksites. In the HD–HC study, the ICCs were considerably smaller, ranging from 0.0004 to 0.003.

## Discussion

These two studies were successful in sampling a multiracial and multiethnic subpopulation of eastern Massachusetts residents. The sampling strategies of both studies reached a subpopulation that is more heterogeneous in racial and ethnic make-up than the greater Boston area. Furthermore, the HD–SB sample has a larger percentage of members with working-class occupations and those with a high school education or less than the general population of adults.

Health disparities in the United States are often described in terms of racial or ethnic inequalities; yet, within racial and ethnic groups, there is variability in both socioeconomic position and morbidity and mortality risk. Nevertheless, populations of color bear a disproportionate burden of poverty (25-27). It is well known that socioeconomic deprivation adversely affects health and increases mortality (12,28). The concepts of social class and socioeconomic position are complex and encompass occupational class, income, poverty, wealth, education, and prestige or status at the individual, household, and area levels (12). We have measured two dimensions of race and ethnicity and three of socioeconomic position. Maintaining these separate characteristics, rather than attempting to define a single measure of socioeconomic position, will allow us to explore the interactions among them in understanding the determinants of successful interventions.

Small percentages of the study sample respondents lived in households that were below the poverty threshold, as expected among a population of working-class participants and those with health insurance. Nevertheless, a substantial proportion of our samples would be eligible for food aid — 15% in the HD–SB sample and 18% in the HD–HC sample. These categorizations do not take into consideration regional differences in cost of living. The greater Boston area is one of the most expensive areas in the country; the self-sufficiency standard for a family of four in that area was $42,564 in 1998 and $54,612 in 2003 (29).

Although both study samples represent multiracial and multiethnic working-class populations, the two samples differ from one another. The HD–SB sample was somewhat younger and included more men than the HD–HC sample. The HD–SB sample had a higher percentage of Asian and Hispanic respondents and a higher percentage of recent immigrants than the HD–HC sample. The HD–HC sample had a higher percentage of participants with household incomes below 185% of poverty than the HD–SB sample. The HD–SB sample has a higher percentage of respondents with less than a high school education than the HD–HC sample.

The differences between the two study samples in the levels of the health behaviors reported by the participants reflect these sociodemographic differences. The workers in the HD–SB sample were more physically active than those in the HD–HC sample were, and a higher percentage of the HD–HC sample members reported taking a multivitamin daily. These differences may be attributable to the differences in age, sex, education, or other factors. Despite these differences in health behavior practices, the percentages of respondents in both samples who were at lower levels of the target behaviors are high, indicating a need for the behavior change interventions in both subpopulations.

The mean hours of physical activity reported by members of both samples is surprisingly high. Although we asked about leisure time activity, participants may have conflated their reports to include activities related to occupational activities, domestic chores, childcare, and walking for transportation, for example. A small validity study done in conjunction with this project indicated, however, that total hours of activity were reported accurately (data not shown).

The response rate to the baseline survey in HD–HC was low, due in part to the fact that potential participants were agreeing to participate in a randomized trial, not just a health survey. This is similar to the low recruitment rate for the businesses in the HD–SB study. The internal validity of the intervention trials is assured by randomization, and the survey is a valid baseline assessment of the levels of behaviors in the two intervention groups prior to intervention implementation. For other researchers who might use the baseline measures and ICCs to plan studies, the generalizability to health center members and workers in worksites who would consent to participate in such a study is also appropriate.  

Planning for group-randomized trials of the effectiveness of interventions targeting modifiable health behaviors requires estimates of the means, variances, and ICCs of the behaviors within the study population of interest (8,10). The estimates reported here apply to four specific health behaviors and two types of randomization groups. The estimated ICCs in the HD–SB sample are similar to those found in other worksite-based intervention studies (30). The estimated ICCs in the HD–HC sample are lower than those in the HD–SB sample are but are similar to those at the district health authority level in England (31). Nevertheless, the ICCs in both studies are sufficient to influence the error variance of the test statistic for evaluating the effectiveness of the intervention and must be included in power calculations for group-randomized studies.

In summary, the procedures developed by these two intervention studies to sample multiracial and multiethnic working-class populations in eastern Massachusetts were successful in identifying such groups. These samples are more diverse in their racial and ethnic make-up and other sociodemographic characteristics than the greater Boston  population. Although the subpopulations resided in the same geographic area and may overlap in other ways, the HD–HC sample was restricted explicitly to exclude anyone in the HD–SB sample. Despite the close proximity of these two subpopulations, they differ in ways that would be expected by their provenance. Furthermore, both samples represent populations with high percentages of members who have cancer-related risk behaviors.

There has been a call for research on the effectiveness of interventions targeting modifiable health behaviors (28), yet intervention approaches have not been designed for or sufficiently tested in working class, ethnically diverse populations (32). Our explicit aim was to recruit from the large multiracial and multiethnic group of working-class men and women at elevated risk for adverse health outcomes. This group is confronted with constraints and limited resources that may influence patterns of health behaviors. We have developed behavioral interventions that respond to the social contextual realities of this group as an approach to reducing the excess burden of cancer borne by communities of color and lower socioeconomic position (4). To fully evaluate the effectiveness of these interventions, it is important to study a diverse population. Future manuscripts will report on the effectiveness of the interventions in promoting change in the behaviors and the influence of the social context on behavior and behavior change.

## Figures and Tables

**Table 1:** Selected Sociodemographic Characteristics of Study Participants in Two Study Samples for a Group-Randomized Trial and of the Population In the Greater Boston Area, 2000–2001

**Characteristic**	**HD–SB^a^**		**HD–HC^a^**		**Eastern Mass CMSA>b**

	**No.**	**%**	**No.**	**%**	**%**

**Sex^c^**					

Male	1170	67.4	747	33.7	47.4
Female	567	32.6	1469	66.3	52.6

**Age, y**					

18-34	445	25.9	318	14.3	d
35-49	758	44.2	757	34.2	d
50-64	451	26.3	788	35.5	d
65+	62	3.6	354	16.0	d

**Race/ethnicity**					

Hispanic	217	12.6	184	8.4	6.8
White	1177	68.1	1291	58.8	80.7
Black	92	5.3	579	26.4	d
Asian	116	6.7	49	2.2	d
American Indian	7	0.4	13	0.6	d
Multiple, including white	49	2.4	47	2.1	d
Multiple, not including white	77	4.5	31	1.4	d

**Birth country**					

Participant not born in U.S.	583	33.6	479	21.7	13.5
Participant born in U.S. but one or both parents not born in U.S.	178	10.3	396	17.9	d
Participant and parents born in U.S.	972	56.1	1336	60.4	d

**Education completed^e^**					

Less than high school	278	16.2	147	6.7	14.7
High school	518	30.2	482	21.9	26.5
Some post-high-school training	578	25.8	537	24.4	23.7
Baccalaureate or more	478	27.8	1033	47.0	35.1

**Poverty index^c^**					

Above 185% of poverty	1320	85.3	1628	82.0	d
Between poverty and 185%	171	11.0	226	11.4	d
Below the poverty line	57	3.7	132	6.6	8.3

**Occupation class^f^**					

Professional, managerial, technical	390	23.7	988	44.9	42.9
Working class	1259	76.3	965	43.9	57.1
No job title	d	d	246	11.2	d

a HD–SB indicates Healthy Directions–Small Business; HD–HC, Healthy Directions–Health Centers.

b Data from the 2000 Census for the Massachusetts part of the consolidated metropolitan statistical area (CMSA) covering eastern Massachusetts (U.S. Census Bureau, 2003).

c Data include individuals aged 18 years and older.

d This characteristic was not selected for comparison.

e Data include individuals aged 25 years and older.

f Data include individuals aged 16 years and older.

**Table 2:** Frequency of Study Participants Meeting and Not Meeting Target Health Behaviors in Two Study Samples for a Group-Randomized Trial, 2000–2001

**Health Behavior**	**HD–SB^a^**		**HD–HC^a^**	

	**No.**	**%**	**No.**	**%**

**Five or more fruit and vegetable servings per day**				

Yes	236	13.7	318	14.5
No	1484	86.3	1882	85.5

**Three or fewer servings of red meat per week**				

Yes	530	30.7	1113	50.6
No	1195	69.3	1088	49.4

**2.5 or more hrs of physical activity per week**				

Yes	1179	72.9	1327	64.7
No	439	27.1	724	35.3

**Multivitamin taken daily**				

Yes	474	27.4	825	37.3
No	1255	72.6	1386	62.7

a HD–SB indicates Healthy Directions–Small Business; HD–HC, Healthy Directions–Health Centers.

**Table 3:** Adjusted^a^ Percentage of Participants Meeting Target Health Behaviors and Intraclass Correlation Coefficients (ICCs) By Study

** Health Behavior**	**HD–SB^b^**			**HD–HC^b^**		

	**No.**	**Adjusted %**	**ICC**	**No.**	**Adjusted %**	**ICC**
Five or more fruit and vegetable servings per day	1720	13.7	0.01	2200	14.5	0.0004
three or fewer servings of red meat per week	1725	31.1	0.02	2201	50.7	0.006
2.5 or more hrs of physical activity per week	1618	72.8	0.006	2051	65.1	0.003
Multivitamin taken daily	1729	26.8	0.02	2211	37.4	0.005

a Adjusted for the clustering of study participants in randomization units (worksites or health centers).

b HD–SB indicates Healthy Directions–Small Business; HD–HC, Healthy Directions–Health Centers.
